# Technological Progress Toward Peanut Disease Management: A Review

**DOI:** 10.3390/s25041255

**Published:** 2025-02-19

**Authors:** Muhammad Asif, Aleena Rayamajhi, Md Sultan Mahmud

**Affiliations:** 1 Department of Plant Pathology, University of Georgia, Athens, GA 30602, USA; 2 School of Environmental, Civil, Agricultural, and Mechanical Engineering, College of Engineering, University of Georgia, Athens, GA 30602, USA

**Keywords:** artificial intelligence, computer vision, disease scouting, drone technology, remote sensing, precision sprayers

## Abstract

Peanut (*Arachis hypogea* L.) crops in the southeastern U.S. suffer significant yield losses from diseases like leaf spot, southern blight, and stem rot. Traditionally, growers use conventional boom sprayers, which often leads to overuse and wastage of agrochemicals. However, advances in computer technologies have enabled the development of precision or variable-rate sprayers, both ground-based and drone-based, that apply agrochemicals more accurately. Historically, crop disease scouting has been labor-intensive and costly. Recent innovations in computer vision, artificial intelligence (AI), and remote sensing have transformed disease identification and scouting, making the process more efficient and economical. Over the past decade, numerous studies have focused on developing technologies for peanut disease scouting and sprayer technology. The current research trend shows significant advancements in precision spraying technologies, facilitating smart spraying capabilities. These advancements include the use of various platforms, such as ground-based and unmanned aerial vehicle (UAV)-based systems, equipped with sensors like RGB (red–blue–green), multispectral, thermal, hyperspectral, light detection and ranging (LiDAR), and other innovative detection technologies, as highlighted in this review. However, despite the availability of some commercial precision sprayers, their effectiveness is limited in managing certain peanut diseases, such as white mold, because the disease affects the roots, and the chemicals often remain in the canopy, failing to reach the soil where treatment is needed. The review concludes that further advances are necessary to develop more precise sprayers that can meet the needs of large-scale farmers and significantly enhance production outcomes. Overall, this review paper aims to provide a review of smart spraying techniques, estimating the required agrochemicals and applying them precisely in peanut fields.

## 1. Introduction

Peanut (*Arachis hypogea* L.), also known as groundnut, is a major cash crop, widely grown in tropical and subtropical regions for its high oil content, making it valuable as food, oil, fodder, and organic fertilizer [1]. Globally, peanut cultivation is widespread, with Asia and Africa accounting for more than 90 percent of the total cultivated area [2]. In the United States, in 2022, the total peanut production was 2.51 million tons, which is approximately 5.1% of the world’s peanut production [3]. Georgia, contributing 54% of the national output, stands as the leading state in peanut production [3]. The significance of peanut as an economic development symbol has grown exponentially, with its market share expanding notably in recent years, partly due to effective disease management [4].

Despite several available disease management techniques, diseases remain a significant constraint on peanut yield, posing substantial production challenges. While numerous traditional practices have been employed, significant yield losses persist, highlighting the necessity for more efficient solutions capable of covering expansive areas with minimal labor while ensuring precise results. Remote sensing technology has emerged as a promising remedy to these challenges, offering real-time, accurate data for disease detection and management across vast agricultural landscapes. The aim of using remote sensing technologies is to develop precise pesticide application methods and equipment specific to peanut crops and their environmental conditions. Remote sensing is a precision agricultural technology that includes collecting, visualizing, and analyzing image data to evaluate crop health at different crop growth stages. The application of remote sensing and AI has been extensively studied in recent years for cost-effective, faster, consistent, and accurate disease identification in different crops [5,6,7,8], including peanuts [9,10]. There are currently several ground-based and aerial remote sensing technologies used to collect image data at variable spatial, temporal, and spectral resolutions. The specific crop growth stage, plot size, and type of sensors are the deciding factors for the selection of the best image resolution for remote sensing [11].

Ground-based sensing systems with different sensors (e.g., RGB, LiDAR, thermal and spectral) offer precise detection and analysis of crop health and structural attributes [12,13,14,15]. Higher-spatial resolution data from ground-based sensing technologies give better distinctions in leaf characteristics and crop area at a stand level. Multispectral imagery helps assess crop health, which visible imagery cannot detect. Similarly, thermal imagery is used to estimate pest pressure, soil moisture, and crop water stress. Microwaves can assess the biophysical characteristics of crops in both day and night conditions and are less susceptible to atmospheric attenuation than visual and infrared remote sensing. Aerial imaging systems also use sensors such as RGB, multispectral, and hyperspectral cameras, which have been widely used for identification of crop diseases. These detection sensors are also effective in identifying morphological and physiological changes in plants, even in uneven terrain and remote areas [16,17,18]. Precision spraying and variable-rate spraying technology offers further progress in precision agriculture, allowing site-specific agrochemical applications on infested parts of the field [19]. By considering canopy morphology, density, and mechanical properties, these technologies improve pesticide efficiency, achieving minimal dosage with optimal droplet deposition and ensuring effective pest control while reducing environmental contamination.

There has been significant progress in remote sensing and advanced sprayer technology applications for disease and pest detection and management in peanut crops. However, no article has reviewed the status of these technologies for peanut production. Over the past decade, numerous review articles have been published on various aspects of peanuts, including traditional disease management technologies [20,21,22], the benefits and effectiveness of using nano-fertilizers [23], factors influencing somatic embryogenesis [24], therapeutic potential and recent trends in medicinal use [25], and evaluating modified cultural practices and cultivation regimes for improving in-row weed control in organic peanut farming [26]. The past literature reviews have largely focused on specific areas, such as disease management through cultural practices and chemical control, breeding, and weed control in peanuts. However, these reviews do not integrate modern technologies, such as remote sensing and AI, into a cohesive system for comprehensive peanut crop health monitoring and targeted spray application for disease management.

This review aims to bridge these gaps by providing an integrated analysis of various advanced remote sensing and AI technologies, focusing on practical applications, environmental considerations, and comparative insights. We perform a thorough review of the published literature and industrial solutions for peanut disease detection and management using aerial and ground-based technologies to explore the advancements made in this area. This review not only delves into the technologies and methods used in the surveyed studies but also critically examines their limitations. This comprehensive approach will offer valuable guidance for researchers, policymakers, and practitioners in precision agriculture, promoting the adoption of advanced technologies for improved peanut disease management. Among the main contributions of this review article are the following:

- Discussing major diseases affecting peanut crops and basic principles of disease management.
- Providing a detailed examination of the advancements in peanut disease identification and detection using imaging and AI techniques implemented through both aerial and ground-based devices.
- Reviewing the latest developments in spraying technologies and evaluating commercial precision sprayers for their effectiveness in peanut farming.

This review paper was written by compiling the recent literature from Google Scholar and renowned journals. The keywords used for finding the literature were ’Smart Sprayer’, ’Peanut’, ’UAV-sprayer’, ’ground sprayer’, peanut diseases’, ’peanut disease management’, ’precision sprayers’, and ’variable rate sprayer’.

## 2. Major Diseases Affecting Peanut Production

Peanuts, a key crop globally, are highly susceptible to diseases, such as early and late leaf spot, sclerotinia blight, southern stem rot, and white mold, which significantly impact yield. Peanut diseases have global distribution variations, attributable to differences in climate, soil conditions, farming strategies, and disease management practices. The primary peanut cultivation areas—the South and North Americas, Asia, and Africa—encounter unique disease challenges that affect output, quality, and economic sustainability. Certain peanut diseases are more prevalent than others because of favorable environments in these areas. High humidity, warm temperatures, prolonged leaf wetness, and high fungicide use offer ideal conditions for early and late leaf spot in the southeastern United States, North America, and Asia. These diseases are managed by growing disease-resistant varieties and applying fungicides in severe cases [27]. Warm days followed by cooler nights, dry weather, and thrips infestation favor the spread of tomato spotted wilt virus and groundnut rosette disease in the South and North Americas and Africa, and they are managed by thrips control and early planting, respectively [28,29]. Drought stress, high temperatures, and high humidity during storage conditions cause aflatoxin contamination in Africa and Asia, which is managed by the application of biocontrol agents [30,31]. Sclerotinia blight is attributed to cool and moist conditions, whereas warm soil temperatures and sandy and loamy soils are good conditions for nematode infestation in the southeastern United States [32,33]. High rainfall in peanut-growing areas of Asia and Africa helps the incidence of rust disease. These diseases are controlled by using resistant varieties and application of fungicides [34].

Fungal diseases are the most common and damaging to peanut crops, affecting all parts of the plant, including seeds, roots, leaves, stems, and pods. Early leaf spot and late leaf spot are the most commonly reported foliar diseases [35]. *Passalora arachidicola* causes early leaf spots, and it is identified by the presence of brown to reddish-brown circular spots on the upper leaf surface, often surrounded by a yellow halo, with light brown spots on the lower surface. *Cercosporidium personatum* is the causative agent of late leaf spots, which manifest as necrotic lesions, premature leaf senescence, and reduced yield [36]. In addition to these, another significant foliar disease affecting peanuts is rust, caused by *Puccinia arachidis*, which features small, reddish-orange pustules on the lower leaf surface, later appearing on the upper surface and impacting seed yield and oil quality [37]. Southern blight, also known as southern stem blight or white mold, is caused by the soil-borne pathogen *Sclerotium rolfsii*. This disease, which affects a wide range of hosts, is characterized by white mycelia covering the plant stem near the soil surface and round brown sclerotia on infected parts [38,39,40]. Cylindrocladium black rot, caused by *Cylindrocladium crotalariae*, leads to field patches, leaf chlorosis, wilting, and stunted growth in peanuts. The fungus infects pegs, pods, and roots, with crimson fruiting bodies visible on diseased branches [39].

In contrast to fungal diseases, viral diseases also pose significant threats to peanut crops. Tomato spotted wilt virus and groundnut rosette disease pose a significant threat to peanuts. Symptoms include concentric ring spots, chlorosis on leaflets, stunting of above-ground parts, small pegs and pods, and reddish seedcoat discoloration with cracking [28]. Immunoassays have confirmed the virus’s presence in field-grown peanut roots, indicating that asymptomatic infections can occur even in genotypes with high levels of resistance to the virus [41].

**Figure 1:**
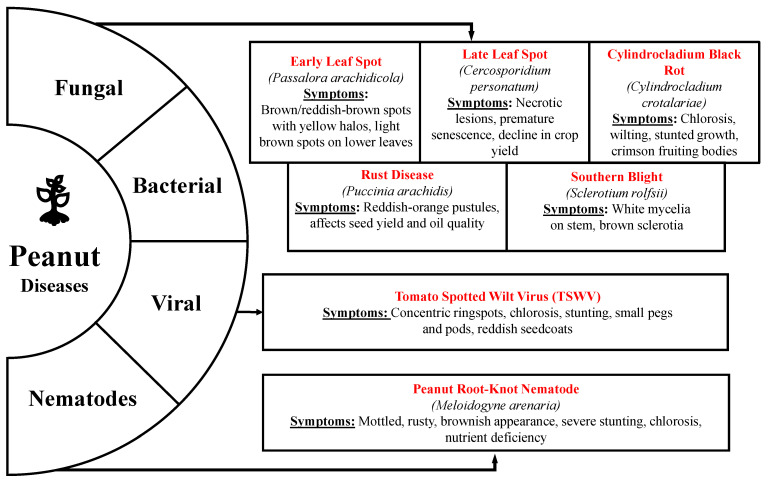
Summary of the major diseases originating due to fungi, bacteria, viruses, and nematodes in peanut crops.

The peanut root-knot nematode (*Meloidogyne arenaria*) is widespread in the southeastern United States. It damages peanut roots, pegs, and pods, leading to severe yield losses of up to 50% in severe cases [42]. This nematode infects a wide range of hosts and thrives in hot, warm climates globally. Infected plants exhibit a mottled, rusty appearance, with above-ground symptoms becoming pronounced at maturity. Symptoms appear in patches in fields, and plants show severe stunting, chlorosis, and nutrient deficiencies. In hot, dry conditions, plant death can occur as the crop nears maturity [43]. Figure 1 summarizes the common diseases caused by fungi, bacteria, viruses, and nematodes in peanuts.

## 3. Basic Principles of Peanut Disease Management

**Figure 2:**
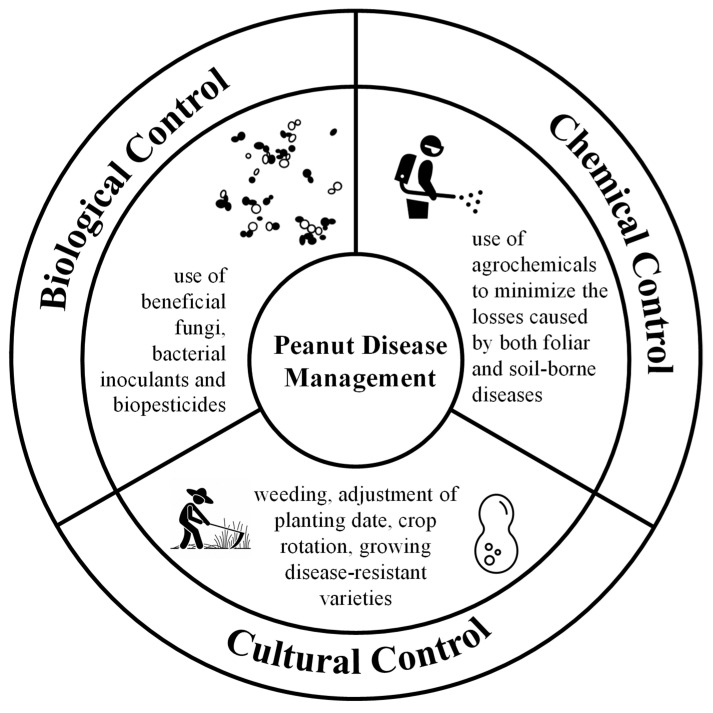
Traditional disease management strategies in peanuts include biological, chemical, and cultural methods.

The basic principles of plant disease control are avoidance of pathogens, their exclusion, eradication, protection, resistance, and treatment. A better understanding of the causes of peanut diseases and the interplay among hosts, pathogens, and the environment helps to develop a range of strategies to control peanut diseases. These traditional disease management strategies include biological, cultural, and chemical control (Figure 2), which continue to evolve and improve as we progress in understanding hosts, pathogens, and the environment [44].

Pests and diseases have long posed significant threats to crops, endangering both agricultural productivity and the livelihoods of farmers. Cultural control methods are essential in peanut farming to mitigate pest and disease pressures without heavy reliance on chemical treatments. Early agricultural practices relied on the genetic diversity of domesticated varieties and landraces, which provided natural resistance to diseases [45]. Cultural methods, such as burning, tillage, and careful site selection, are among the earliest strategies used to manage crop diseases [46], which are primarily preventive and minimize the disease risks. Some other methods include strategic crop rotations with non-host plants like grains or legumes to disrupt pest cycles, optimal timing of planting to avoid peak disease periods, and selecting resistant peanut varieties [26]. Recent studies indicate that incorporating rotations with bahiagrass further reduces disease incidence and leads to increased crop yields. Specifically, peanut yields were shown to increase by 19% following two years of corn rotation and by 41% following two years of bahiagrass rotation [47]. The disease incidence can be avoided by crop rotation, the application of insect traps, mechanical removal of infected plants by machinery or manual operations, adapting certain planting dates to elude disease outbreaks, and using appropriate tillage and soil preparation methods [48]. Moreover, effective weed management and sanitation practices help reduce disease sources, while maintaining soil health through proper irrigation and nutrient management supports plant vigor and disease resistance. However, challenges include the need for continuous monitoring and adapting practices to local conditions, alongside limitations in eliminating all pests and diseases without some chemical intervention.

The biological control of peanut diseases employs helpful fungi, bacterial inoculants, and biopesticides for disease management. *Trichoderma* spp. are found to be effective against *S. rolfsii*, which causes stem rot in peanuts [49]. Similarly, *Paecilomyces lilacinus* is a fungus that is applied to help control nematode diseases in peanuts by eradicating the eggs and juveniles of nematodes [50]. Some strains of *Bacillus* spp., i.e., *Bacillus circulans* and *Serratia marcescens*, produce antibiotics and induce systemic resistance in plants against fungal growth [51]. The biopesticides extracted from micro-organisms such as *Trichoderma*, *Phytophthora*, and *Bacillus thuringiensis* are used as biofungicides, bioherbicides, and bioinsecticides, respectively. They are effective in managing pests and diseases in peanuts with minimum impact on non-target plants and less stress on organic farming to provide food commodities free of toxic residues. Biopesticides offer a safe alternative to chemical control, and help lessen the long-term risk of pesticides on human health and the environment [52]. Despite their benefits, their effectiveness decreases with multiple pathogens, they can be costly initially, and environmental factors like temperature, soil conditions, and humidity influence them. Their effectiveness may also decrease with changing pathological strains [53,54]. Other challenges include optimizing application methods, ensuring efficacy across varied environmental conditions, and integrating them effectively into farming practices to maximize disease control while minimizing ecological impact. There is also the chance of the crop being infected even after adopting preventive measures against the disease, and then we have to depend on chemicals to minimize the financial loss [55].

Chemical control remains the most widely employed method in peanut farming due to its proven effectiveness and immediate action against pests and diseases. Chemical products constitute the second most significant expense in peanut crop cultivation, following certified seed [56]. Though management practices vary in different regions of peanut production, multiple applications of agrochemicals are common to minimize the losses caused by foliar and soil-borne diseases [57]. Among the various agrochemicals available on the market for the management of peanut diseases, some include chlorothalonil combined with sulfur or copper, which is applied for foliar diseases at 10 to 14 day intervals; azoxystrobin, effective against southern stem rot; and the fumigant metam sodium, recommended for Cylindrocladium black rot. While traditional methods have proven effective, there is increasing interest in enhancing the efficiency and sustainability of agrochemical applications. The broadcast method, commonly used in peanut farming, often leads to substantial pesticide use. However, precision band sprayer systems equipped with AI vision sensors offer a more targeted approach, reducing chemical waste by accurately assessing the peanut canopy and applying agrochemicals based on canopy volume [58].

Despite their widespread use and utility, pesticides carry a substantial risk when not applied precisely or overused. They can lead to the development of chemical resistance in peanuts, potentially causing diseases to reoccur and making the pesticides ineffective. Moreover, pesticides harm non-targeted plants and pose significant risks to both the environment and human health. However, advancements in agricultural technologies, such as precision boom sprayers or variable-rate sprayers with integration of AI and remote sensing, enable more precise pesticide application and targeted management of diseased plants. These advanced spraying techniques are increasingly important as they allow for targeted application of pesticides, reducing over-spraying and minimizing environmental and health impacts [59]. By precisely targeting areas affected by pests and diseases, farmers can optimize pesticide use, minimize chemical residues in the environment, and mitigate the development of pesticide resistance [60]. This approach supports more sustainable agricultural practices while maintaining effective pest management strategies.

## 4. Ground-Based Sensing for Peanut Disease Scouting

The advancements in agricultural technology and the growing need for efficient and sustainable farming have led to the application of these technologies (e.g., computer vision, AI, remote sensing) for crop disease detection and management over traditional clinical methods [61]. Ground-based sensing technologies using camera sensors, such as RGB (red–green–blue), multispectral, hyperspectral, and thermal cameras, are effective in identifying diseases in peanuts. These vision sensors can detect peanut foliar diseases, including peanut leaf spots, leaf wilting, peanut southern blight, and stem rot. The sensors used in these ground-based peanut disease detection systems achieve optimum accuracies at specific wavelengths [62].

RGB camera sensors are cost-effective imaging devices capable of efficiently detecting and pinpointing disease symptoms in visible light (400–700 nm) in three spectral bands: Red, green, and blue. These sensors work on the principle of color differentiation, leveraging variations in the color, texture, and geometric shape (curling, spots, defoliation) of leaves [63]. These sensors facilitate faster, cost-effective, automated, consistent, AI-driven, and large-scale disease detection in real-time scenarios.

RGB channels facilitate the identification of disease-induced changes, including chlorosis (yellowing), necrosis (browning), and lesion development on leaves. The images are later analyzed using computer vision and machine learning algorithms to differentiate between healthy and diseased plant parts [64,65]. High-throughput phenotyping (HTP) using RGB images is useful in efficient data collection and analysis in peanut breeding [66]. RGB cameras like the Sony α600 (Sony Corporation, Tokyo, Japan), Samsung NX300 digital camera (Samsung Electronics, Suwon, South Korea), Canon EOS Rebel T6i (Canon Inc., Tokyo, Japan), Olympus Imaging Corp C7070WZ camera (Olympus Corporation, Tokyo, Japan), and Canon EOS Kiss X5 camera(Canon Inc., Tokyo, Japan) have been used for high-throughput phenotyping of groundnut rosette virus in peanut breeding; detection of leaf wilting with 90% accuracy; detection of tomato spotted leaf virus, late leaf spot, and hyperburn injury with 86% accuracy; and development of a rapid online recognition method for peanut foliar disease symptoms like black spot, brown spot, net spot, and mosaic disease [67,68,69,70]. In peanut breeding programs, advancements are aimed at enhancing moisture stress tolerance, where leaf wilting serves as a critical indicator. To efficiently collect data across numerous peanut lines, breeders utilize various color space indices such as intensity, hue, saturation, etc., derived from RGB images. This method replaces labor-intensive visual assessments and ensures precise measurements of leaf wilting to support more effective breeding strategies. This emphasizes using innovative RGB-derived models for precise leaf wilting estimation in peanut genotypic screening under water stress. RGB images can discriminate between turgid and wilted plants by detecting changes in canopy color due to water deficiency, shifting from green (120° hue) to yellow (60° hue). This aids in efficient irrigation scheduling. Additionally, image analysis and regression models are effective diagnostic tools for peanut foliar diseases. Farmers can access these diagnostic algorithms through mobile applications or web platforms; they offer up to 98.7% accuracy in disease identification [69].

Hyperspectral cameras have also been used for peanut disease scouting. Measuring hyperspectral reflectance from peanut canopies has made disease scouting and forecasting possible at early or asymptomatic stages. Hyperspectral cameras capture more spectral bands than RGB sensors, hence they ensure better differentiation of disease symptoms. They are also very applicable in the assessment of nutrient deficiencies. The canopy reflectance in the near-infrared (NIR) regions decreases as the severity of peanut leaf spot disease increases. The NIR range of 750–950 nm has a considerable impact on canopy reflectance, which makes it a reliable method for image-based early detection of leaf spot infestation in peanuts. Hyperspectral sensors combined with machine learning can distinguish between symptomatic and healthy peanut plants by identifying the key wavelengths [71]. Hyperspectral sensors, like the ASD Field Spec3 spectrometer (ASD Inc., Boulder, CO, USA), have been used to collect the spectral data of peanut leaves at three key wavelengths—570 nm, 671 nm, and 750 nm—to identify peanut leaf spot disease symptoms at 96.88% accuracy and assess southern blight severity at 90.5% accuracy [72,73]. The Jaz spectrometer has been used with machine learning models to identify healthy and diseased plants in peanut stem rot detection at over 90% accuracy using two-class classification at the optimal wavelengths of 501–505 nm, 690–694 nm, 763 nm, and 884 nm [74]. Thus, using hyperspectral sensors to monitor peanut southern blight notably improves disease management and control strategies.

Handheld spectrometers are portable devices that work on the principle of spectroscopy to analyze light absorption, reflection, and transmission in plant tissues. They capture reflectance information from visible and near-infrared spectral regions and are applied to identify peanut diseases. These sensors collect precise data on water content, plant pigments, and stress levels even in low-light conditions. These spectrometers measure the severity of early and late leaf spots by measuring canopy reflectance in the 520–600 nm and 700–850 nm regions [75].

Thermo-vision cameras, like Fluke Ti 32 (Fluke Corporation, Everett, WA, USA), are infrared thermal imaging sensors that detect thermal radiation emitted by the plant surface. They are used in the detection of plant disease and stress monitoring even before the symptoms appear on plant surfaces. Thermal imaging offers a powerful and non-invasive tool for peanut disease scouting, detecting subtle changes in plant temperature that can predict disease incidence. The leaf temperature decreases by 1.3 °C before the leaf spot symptoms appear on the peanut crop and then it increases by 2.2 °C compared to the healthy plants after the plant is affected by the disease. This temperature difference distinguishes the healthy plants from the diseased plants and predicts the onset of early leaf spot and late leaf spot with overall accuracies of 78% and 89%, respectively. Thermal cameras can be used for early detection of stomatal closure because of temperature changes caused by environmental stress or pathogenic infection. This allows farmers to take timely preventive actions [75].

The traditional methods used for disease identification and phenotyping are being replaced by state-of-the-art data collection tools, machine learning algorithms, and remote sensing technologies. RGB cameras provide high-resolution visual signatures for evaluating apparent symptoms, whereas hyperspectral cameras and spectrometers acquire intricate spectral signatures beyond human perception, facilitating the detection of biochemical and physiological changes linked to biotic and abiotic stress. Thermal imaging cameras improve disease detection by detecting temperature fluctuations associated with plant transpiration and stress reactions. By using these sophisticated imaging tools, researchers and farmers can enhance disease management strategies, facilitating prompt interventions and minimizing crop losses.

**Figure 3:**
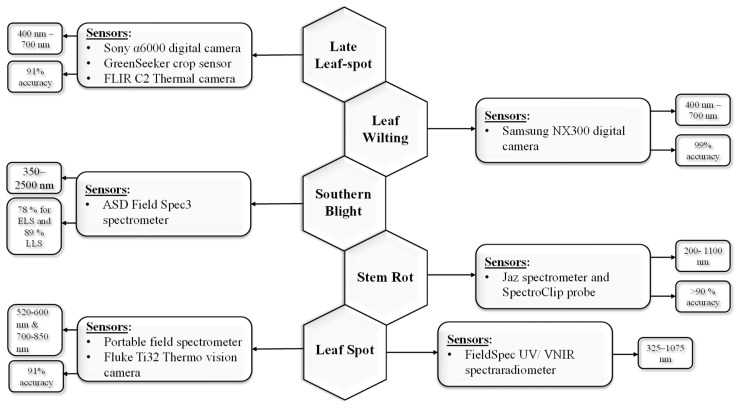
A diagram visualizing a summary of different types of ground-based disease scouting sensors along with their wavelengths for common peanut diseases from studies published in the last decade.

Table 1 provides a comprehensive overview of the diverse ground-based sensing technologies employed in the last decade in the detection and diagnosis of various diseases affecting peanut crops, and Figure 3 summarizes this information visually.

## 5. UAV-Sensing-Based Peanut Disease Scouting

Ground-based sensing methods, although widely used, are limited in their coverage area and spatial resolution. UAVs are widely employed in agriculture for crop management to circumvent these restrictions. A UAV can be operated remotely, and it is equipped with a multi-sensor imaging system that includes RGB, multispectral, or hyperspectral cameras for autonomous data collection from the crop through high-resolution spatial and temporal images. UAVs are commonly used in agriculture to estimate crop health, especially in hard-to-reach areas, by scanning many acres in minutes. These sensors measure the vegetative indices such as the Normalized Difference Vegetation Index (NDVI), Green Normalized Difference Vegetation Index (GNDVI), and Excessive Green Index (EGI) to accurately assess plant biomass and identify diseases, water stress, and nutrient deficiencies [76]. Some of the recent studies mentioned below have utilized UAV remote sensing technologies in peanuts to enhance disease detection capabilities.

RGB cameras have become an essential component of UAV-based remote sensing systems for peanuts, offering a practical solution for real-time monitoring at high resolution. The recent rise of highly accurate yet low-cost RGB sensors and computer vision support has facilitated their application in the identification and diagnosis of peanut diseases [77]. These sensors capture light reflectance in multiple bands through aerial RGB images for the identification of peanut disease symptoms. RGB images collected by the Sony^®^ α6000 camera mounted on an octocopter UAV AscTec^®^ Falcon 8 (Ascending Technologies, Krailling, Germany) have been used in estimating peanut leaf wilting with 90% accuracy [68]. A similar sensor had also been used in assessing the NDVI and other matrices like the Green Area (GA) and Greener Area (GGA) to correlate them with the peanut yield in case of potential disease incidence or abiotic stress [78]. UAV-derived vegetation indices, including those from RGB images, are valuable tools in predicting diseases and yield, highlighting the potential of UAV-based imaging for early yield estimation in peanut breeding programs. While there is limited literature on managing peanut diseases using RGB cameras, these cameras have been effectively employed for disease diagnosis in other crops.

UAV-based spectral imaging in peanuts harnesses leaf-level reflectance to establish vegetation indices for detecting bacterial wilt disease. A FieldSpec 3 Hi-Res spectroradiometer camera (ASD Inc., Boulder, CO, USA) and a Parrot Sequoia multispectral camera (Parrot S.A., Paris, France) mounted on a Parrot Bluegrass Drone (Parrot S.A., Paris, France) have been used to identify the most useful hyperspectral indices to distinguish between healthy and symptomatic plants for bacterial wilt disease in peanuts, even with very few symptoms [79]. Healthy plants exhibit the highest leaf reflectance values, which decrease gradually in the visible and NIR regions as disease progresses due to reduced water and chlorophyll contents. Different diseases have certain effective wavelength ranges for effective detection, and for detecting bacterial wilt in peanuts, the recommended wavelength range is 700–900 nm, with the most effective wavelengths being 730 nm and 790 nm [80]. Drone-based multispectral imagery can also effectively quantify defoliation in peanut plants [81], which is usually marked by the symptoms of defoliation and lesions affected by leaf spot disease [36]. A multispectral MicaSense RedEdge sensor (Micasense, Seattle, WA, USA) mounted on a Phantom 3 Advanced multi-rotor drone (DJI, Shenzhen, China) and DJI Phantom 3 quadcopter (DJI, Shenzhen, China) in another study was very accurate in identifying six vegetation indices, i.e., Normalized Difference Red Edge (NDRE), Soil Adjusted Vegetation Index (SAVI), Difference Vegetation Index (DVI), Leaf Area Index (LAI), Simple Ratio (SR) index, and Reduced Simple Ratio Vegetation Index (REVI), that highly correlate with the late leaf spot disease rating at the late growth stage of peanuts [82,83]. Hence, the use of UAVs not only reduces labor costs but also ensures more consistent results compared to traditional manual inspection methods.

Hyperspectral imaging is not efficient for broader assessments like multispectral images, but it provides more in-depth information for cutting-edge research about disease identification based on the unique spectral fingerprints of disease symptoms. It can collect data in hundreds of narrow, contiguous spectral bands that provide highly accurate spectral information on a pixel level [84]. A hyperspectral imager GaiaSky-mini (Gaiasky Tech Co., Ltd. Shenzhen, China) mounted on a Matrice 600 UAV (DJI, Shenzhen, China) was used to select 586 regions of interest (ROIs) with different severity levels of peanut leaf spot disease. This helped lead to peanut leaf spot disease identification at 90.29% overall accuracy [85].

Polarization-sensitive cameras also effectively distinguish between healthy and diseased plants by their changed polarization signatures; they detect structural and biochemical changes associated with plant diseases for accurate disease scoring. Polarization imaging offers non-spectral-based disease identification based on the unique surface properties of the symptomatic plants [86]. UAV-based polarization imaging captures the differences in how light reflected from plant surfaces interacts with healthy and diseased plant tissues. A polarization-sensitive Triton 5.0 MP camera (Lucid Vision Labs, Inc. Richmond, BC, Canada) mounted on a DJI Inspire 2 drone (DJI, Shenzhen, China) was used to collect the polarization signatures of peanut plants that were defoliated as a result of peanut leaf spot disease in North Carolina [87].

These aforementioned studies show the importance and applicability of UAV-based sensing for peanut disease scouting, especially in terms of disease forecasting and management. UAV-assisted disease surveillance and advanced data processing techniques like neural networks and spectral indices enhance accuracy and efficiency in disease identification. Remote sensing technologies enable large-scale field monitoring, surpassing manual inspection in speed and coverage. These methods can significantly control the spread of infectious diseases in agriculture by enabling precise and objective evaluation of disease severity. Integrating UAV-based remote sensing with machine learning and geographic information systems (GISs) enables farmers and researchers to enhance disease surveillance, optimize pesticide application, and improve crop health management.

**Figure 4:**
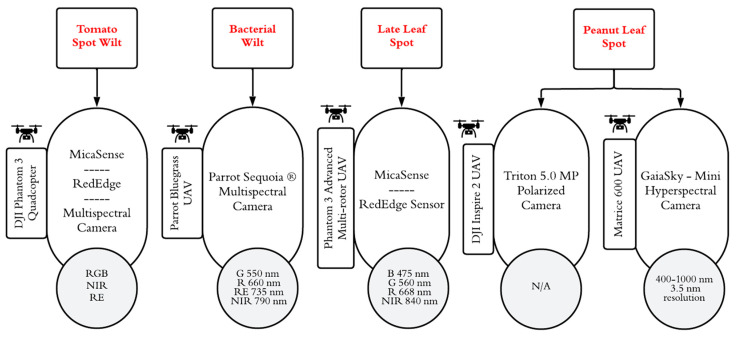
A diagram visualizing a summary of different types of UAV-based disease scouting sensors for common peanut diseases from published studies in the last decade.

Table 2 provides a comprehensive overview of the diverse UAV-based sensing technologies, along with their effective wavelengths, measuring platforms, detection approaches and models, and limitations, employed in the last decade in the detection and diagnosis of various diseases affecting peanut crops, and Figure 4 summarizes this information visually.

## 6. Ground Sprayer Technology for Peanut Disease Management

Ground sprayers are the primary choice for managing peanut diseases, widely favored in row crops due to their maneuverability and coverage capability across the field. Available in various sizes and configurations, ground sprayers are available in ranges suitable for diverse crop types, terrains, and application requirements. While conventional sprayers have made significant advancements in the past decade, including broader application widths and higher speeds that enable more acres to be covered per tank load, they still lag in precision and variable-rate spray application [88]. Key technological advancements in ground sprayers are focused the nozzle type, spray pattern, pump system, tank capacity, control mechanism, boom structure, and compatibility with precision or variable-rate spraying technology, which includes the application of sensors and predefined mapping tools for the application of pesticides at a precise or variable rate across the field [89].

Hydraulic knapsack sprayers are widely recognized handheld tools used for pesticide applications. Although commonly employed in small areas for spot treatment, they are extensively utilized across diverse crops, including peanuts, in tropical regions [90]. These manually operated sprayers function with the assistance of a hand lever to maintain constant pressure and usually have a tank capacity of 15 L [91]. Backpack sprayers are the most widely used sprayers in small-scale peanut farming. They have been used in peanut research for applying plant growth-promoting rhizobacteria (PGPR) to study the induced systemic resistance against late leaf spot disease caused by PGPR, azoxystrobin and tebuconazole, nitrogen and zinc, sulfentrazone, and chlorothalonil [92,93,94,95,96].

Although backpack sprayers are effective for small-scale spray applications, they lack reliability for larger operations. In contrast, boom sprayers are highly convenient and efficient for uniform, large-scale spraying. The consistency of the spray application is influenced by the movement of the spray boom, while the spray rate is determined by the nozzle output, the boom’s longitudinal speed, and the overlap of the spray nozzles, which is, in turn, affected by the boom’s instantaneous height [97]. Boom sprayers have been used in different studies to determine the efficacy of fluazinam, procymidone, and iprodione at different rates for the control of sclerotinia blight in peanuts and to assess the effect of the rate and application interval of pyraclostrobin on the management of early leaf spot [98,99]. A boom sprayer was also used in a study that involved the application of benomyl and chlorothalonil to compare the application strategies of fungicide-resistance management against late leaf spots of peanuts [100]. Commercial boom sprayers have been used to study the effect of droplet size on pest control in peanuts, with the study concluding that the nozzle type did not affect pest control as coarse-droplet nozzles were equally as effective as fine-droplet nozzles [88]. The study indicated that air-induction nozzles are efficacious for pesticide applications in both peanuts and cotton, maintaining insect control and yield integrity.

Variable-rate spraying or precision spraying is a sophisticated precision agriculture technique that modifies spray application rates in real-time according to field conditions. It optimizes the pesticide application with enhanced spray efficiency and environmental sustainability [101]. An IC35 rate controller (TeeJet Technologies, Logan, UT, USA) and a DynaJet 7140 PWM system (TeeJet Technologies, Logan, UT, USA) were used in a study to evaluate the efficacy of two flow control systems—rate controller (RC) and pulse-width modulation (PWM)—on an agricultural sprayer in simulated site-specific application situations. The findings demonstrated that PWM systems exhibit greater responsiveness and efficacy, rendering them the optimal selection for precise pesticide applications [102]. Another study used a Micro-Trak MT-2405F II rate controller (Micro-Trak, Mankato, MN, USA) to evaluate the effects of different ground speeds on spray deposition and quality, utilizing a commercial agricultural boom sprayer. The sprayer with a rate controller provided more uniform and efficient spray deposition than traditional sprayers, especially when the ground speed fluctuated during the pesticide application [103]. Hence, ground-based sprayers offer a wide range of specifications based on their applicability and required efficiency. Backpack sprayers, boom sprayers, and variable-rate sprayers fulfil distinct functions in agricultural practices: backpack sprayers are ideal for small-scale or targeted treatments, boom sprayers provide consistent coverage across fields, and variable-rate sprayers enable precise application according to real-time field conditions. Variable-rate sprayers provide the highest efficiency and precision.

**Figure 5:**
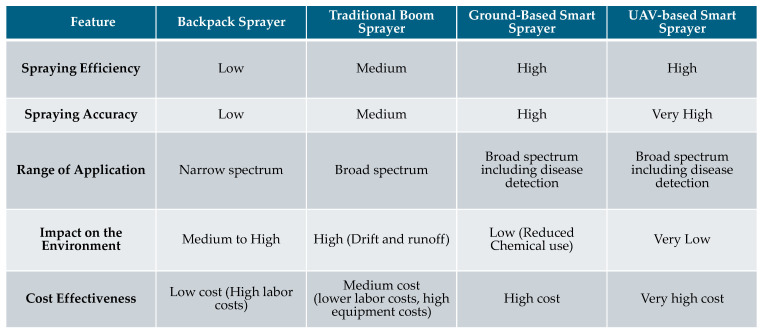
Comparison of four types of sprayers on five different parameters.

Table 3 provides a comprehensive summary of different types of ground-based sprayers, their performance along with the chemicals used, and limitations focused on different peanut diseases from published studies of the past decade, and Figure 5 summarizes this information visually.

Figure 5 below shows a comparison of backpack sprayers, traditional boom sprayers, ground-based smart sprayers, and UAV-based smart sprayers against five different efficiency parameters. Backpack sprayers and boom sprayers are less costly; they are less efficient and cause more spray drift. In contrast, ground-based and UAV-based smart sprayers offer very efficient spray application with minimum environmental impact, although they are relatively expensive.

## 7. UAV-Sprayer Technology for Peanut Disease Management

Despite the advantages, ground-based spraying systems face challenges such as low coverage and efficiency and the potential for crop damage. UAV-sprayers with smart decision-making capability offer high efficacy, labor savings, and the ability to perform mechanical plant protection operations without crop damage [105,106]. Moreover, UAV-based sprayers offer several advantages over conventional sprayers, including faster spraying speeds (3–7 m/s), variable application altitudes, and the production of smaller droplets [107,108].

There are various types of UAVs, depending on their size, lifting capacity, power type, and configuration, and they range from fixed-wing to single- and multi-rotor aircraft. The most common UAVs used for agricultural spraying, such as for peanuts, are multi-rotor and electrically powered [109]. They have become a popular choice for agrochemical applications because of their outstanding flight stability, efficient spray application, and convenient maintenance [110]. Shan, Wang [111] used a T20 plant protection UAV (DJI Technology Innovation Co., Ltd., Shenzhen, China) to apply four different pesticide doses to control peanut leaf spot, rust, and leaf defoliation. They compared the deposition characteristics of spray on the peanut canopy and ground using a 3WBD-16A electric knapsack sprayer (Taizhou Luqiao Qili Agricultural Machinery Co., Taizhou, China), and the results showed that the UAV-based sprayer had a higher droplet density and spray coverage, by 87.5% and 90.0%, respectively.

Suspension concentrates are among the most frequently utilized formulations in agricultural plant protection. However, in the rapid advancement of UAV technology in agricultural spraying systems, issues such as spray drift, inadequate wettability, and droplet rebound are prevalent. Wang, Li [112] demonstrated that the addition of tank additives, referred to as adjuvants, can enhance control efficacy and extend the duration of pesticide effectiveness in UAV-based plant protection. Sun, Zhao [107] used the EA-20X UAV sprayer (Eavision Technologies Co., Ltd., Suzhou, China) to study the effects of tank-mix adjuvants on the dosage delivery of suspension concentrates on hydrophobic peanut leaf surfaces. The results demonstrated that the MO501 adjuvant significantly inhibits the droplet rebound on peanut leaves at concentrations of 0.5% or higher and Silwet 408 achieves complete wetting and superspreading at 0.2% or greater concentration.

According to Faiçal, Freitas [113], high accuracy in pesticide spraying is achieved by the AdEn (Adaptation to the Environment) system, which automatically adjusts UAV controls according to changing weather conditions, particularly wind direction and speed. This is important in peanut cultivation because it ensures precise pesticide application with minimum spray drift and environmental impact.

Similarly, UAVs have been applied in many row crops other than peanuts to improve the spray application. For instance, Qin, Xue [114] studied the droplet deposition and efficiency of fungicides sprayed with an N-3 helicopter to control powdery mildew in wheat. This study focused on the impact of UAV working height and different spraying concentrations on droplet deposition on the wheat canopy and disease control. The performance of the UAV was significantly better than that of the electric knapsack sprayer in terms of droplet coverage on the lower leaves of wheat infected by powdery mildew. Likewise, Lou, Xin [115] used a Jifei P20 quadrotor (Jifei Technology Co., LTD, Guangzhou, China) at different heights to elucidate the effects of UAV flight height on spray droplet distribution and spray drift for controlling aphids and spider mites in cotton. They discovered that droplet uniformity, canopy coverage, and droplet deposition were optimal when the UAV was flown at a flight height of 2 m.

Precision and variable-rate spraying approaches are emerging technologies with significant potential for efficient pesticide use, adjusting the flow rate based on factors such as aircraft speed, height, and plant density within specific sections of the field [116]. This is an advantage over conventional spraying, which typically applies pesticides uniformly across the entire field regardless of variations in plant density or other conditions. AI paired with UAV technology represents a transformative force in precision agriculture. For example, Wen, Zhang [117] combined plant protection UAV operations with artificial neural network (ANN) technology and trained an error backpropagation (BP) model to control droplet deposition. Based on the real-time information collected by multi-sensors, plant protection UAVs deposit the agrochemicals on the crop at variable rates to ensure uniform deposition in constantly varying factors like ambient temperature, humidity, wind speed, flight speed, and altitude. The predicted deposition amount thus regulates the flow rate of the spray system based on the field condition. UAVs may overlook sections of the field during spraying operations. Wireless sensor networks (WSNs) employed in UAVs optimize the flight path to reduce the likelihood of the aircraft sprayer neglecting any area of the field [118].

UAV-based sprayers are becoming increasingly important for the precise application of agrochemicals in managing peanut diseases. These systems offer a highly efficient and cost-effective solution for large-scale pesticide application, especially as climate change exacerbates the risk of disease outbreaks [119]. By integrating UAV technology into crop management practices, farmers can achieve more accurate and targeted spraying, reducing chemical waste and environmental impact. This advancement not only modernizes agricultural practices but also supports sustainable farming by adapting to the challenges posed by a changing climate.

Table 4 provides a comprehensive summary of different types of UAV-based sprayers along with their specifications, focused on different row crop diseases from published studies of the past decade.

## 8. Commercial Precision or Variable-Rate Sprayers in Disease Management

The integration of advantaged scouting technologies is essential to ensure sustainable agricultural practices that can cope with the increasing global food demand and the challenges posed by climate change [124]. Some studies have integrated precision technologies and implemented them to develop precision commercial sprayers. For instance, Sassu, Ghiani [125] developed a small pesticide spraying system (<1 kg payload) for the Phantom 4 Pro DJI drone (DJI, Shenzhen, China), enhancing its application capability of commercial bio-pesticides on horticultural crops. This system utilized low-cost components and included an electric pump, XR nozzles for precise spraying, and a remote activation system. Field tests demonstrated effective pesticide distribution and flight autonomy despite challenges like wind interference and payload limitations. The sprayer’s ability to perform ultra-low-volume applications underscores its potential in agriculture, offering advantages over traditional methods through precise application and reduced operator exposure to chemicals. Similarly, Auburn University’s research on the DJI Agras T40 spray drone (DJI Technology Co., Shenzhen, China), costing USD 35,000 to USD 55,000, highlights its efficiency in spraying crops on wet fields and difficult terrain, and in reducing fuel use, though it faces limitations like battery life and signal loss, making it a cost-effective but complementary option to conventional methods [126].

Advancements in drone spraying technology are advancing at a rapid pace. Notably, eVTOL (electric vertical takeoff and landing) drones have emerged, demonstrating significant capabilities in precision agriculture. These innovative drones are poised to revolutionize agricultural practices by offering enhanced efficiency and accuracy in the application of pesticides and fertilizers. For instance, theSC1 crop dusting drone (Guardian Agriculture, Woburn, MA, USA), the first FAA-approved commercial eVTOL in the U.S., efficiently sprays up to 60 acres per hour with a 200-pound payload. It features a combined tank fill and supercharge time of less than one minute [124]. A T20 DJI plant protection UAV sprayer (DJI Technologies Inc., Shenzhen, China) and a 3WBD-16A electric knapsack sprayer (Taizhou Luqiao Qili Agricultural Machinery Co., Taizhou, China) were used to evaluate the impact of application volume rates and pesticides on droplet deposition, disease incidence, leaf retention rate, and peanut yield. The UAV sprayer, with an application volume rate of 22.5 L ha^−1^, exhibited a higher control effect on the peanut defoliation rate, leaf spot, rust disease, and peanut yield, demonstrating just a 0.3% lower efficacy in managing leaf spot compared to the electric knapsack sprayer [111]. This supports the viability of employing plant protection UAVs to manage peanut diseases for better crop quality and yield.

However, there is an increasing demand for commercial ground-based and UAV-based precision sprayers for peanuts. These sprayers need to be designed according to the unique characteristics of the crop to effectively manage large peanut fields and help farmers meet market requirements. Furthermore, it is essential to develop farmer-friendly commercial sprayers that are readily available and adaptable for farmers of all scales. While advancements in precision disease scouting and sprayer technology are significant, the growing population and evolving market need to emphasize the urgency for the development and availability of more commercial sprayers in the future.

In Table 5, a detailed overview is provided of various commercial precision sprayers that have been introduced and applied in pesticide management across different agricultural crops, as documented in studies published over the past decade.

## 9. Discussion and Future Perspectives

Despite the significant potential of advanced peanut disease management technologies, several challenges must be addressed to maximize their effectiveness. Modern agricultural equipment, such as large-scale ground sprayers, continues to face significant challenges. One of the primary concerns is the risk of cross-contamination, where the equipment can transfer pathogens from diseased to healthy areas. Although these sprayers are highly efficient at covering large areas, they can inadvertently spread diseases like white mold by carrying pathogens across the field, particularly in dense plant populations [40]. Additionally, traditional spraying methods often struggle to reach the basal regions and canopy of peanut plants, leading to suboptimal coverage and leaving lower parts of the plant vulnerable to disease [134]. Adding an airflow unit to the existing smart ground-based boom sprayers might help deposit droplets in the basal regions and near the roots, aiding in the management of root and soil-borne peanut diseases. Using precision ground-based sprayer systems for peanuts involves some additional challenges. For instance, when the ground has dried enough to permit the use of large ground sprayers, these machines can still cause considerable soil compaction, which may result in decreased crop yields [135]. Moreover, large-scale ground sprayers must be designed to minimize soil compaction while maintaining effective coverage. Ensuring these technologies benefit all peanut farmers requires efforts to improve technological literacy and accessibility. Educating farmers on the benefits and operational intricacies of smart sprayer technologies and advanced sensors can promote widespread technological adoption.

UAV-based sprayers equipped with rotary atomizers or fans present a promising solution. These sprayers can deliver a fine, uniform spray to the lower parts of the plant and the soil surface, which is critical for controlling diseases like white mold. Peanuts have a unique growth habit, with a low canopy and spreading root system, making adequate coverage of the lower plant parts and soil surface critical [136]. Rotary atomizers or fans provide a fine, uniform spray that reaches these hard-to-access areas and is more suitable for low-volume and variable-rate spraying, both of which are not possible by hydraulic nozzles [137]. This approach enhances disease and pest control, improves nutrient uptake, and improves overall crop health and yield. Fang et al. [138] reviewed and compared hydraulic nozzles and rotary atomization sprayers used in UAVs for plant protection, concluding that rotary atomization sprayers are more effective for low-volume and variable-rate applications, making them the preferred choice for future UAV and robotic sprayer designs. Moreover, UAVs’ ability to perform low-volume and variable-rate spraying enhances their suitability for precision agriculture, allowing for targeted agrochemical applications.

To further improve the effectiveness of UAV-based sprayers, retrofitting them with additional sensors, such as wind sensors, can significantly mitigate issues like spray drift, where the wind carries pesticide sprays to unintended areas, resulting in pesticide loss and potential environmental and public health risks. Wang et al. [105] quantified the effect of wind speed and pesticide droplet size on the UAV spray drift to develop a protocol drift risk assessment. By measuring wind speed and direction and using these data to adjust spray applications in real-time, UAV systems demonstrated a lower drift distance compared to traditional methods. Application precision can further be enhanced by incorporating a modeling approach into these UAVs that considers environmental factors like wind, altitude, and pesticide spray characteristics, such as velocity and droplet diameter. Additionally, equipping UAVs with integrated edge computing devices, which are AI-powered, embedded systems that analyze data at the point of collection, or utilizing UAVs as edge computing platforms, can enable real-time pesticide deposit calculations, optimizing spray accuracy even further.

UAV-based sprayers also face limitations, including limited payload capacity, short battery life, susceptibility to extreme weather, and government clearance requirements [139]. These factors complicate the deployment of UAV-based systems, particularly in time-sensitive situations like disease outbreaks. To overcome these challenges, future research should be focused on developing UAVs with improved battery life, enhanced weather resistance, and greater payload capacities. Integrating advanced AI algorithms with UAV systems can also refine disease detection and spraying accuracy, reducing the likelihood of disease spread and ensuring more effective crop protection.

Looking ahead, continuous advancements in sensor technology and the utilization of ground-based and UAV-based sprayers hold promise for refining disease management strategies in peanuts. To maximize peanut production and minimize yield losses, future research should focus on integrating these advanced technologies into holistic management systems, exploring synergies between smart sprayers, advanced sensors, and precision agriculture techniques. While challenges exist in adopting and refining these methods, proactive measures can pave the way for the effective deployment of advanced disease management practices to optimize production efficiency for sustainable peanut yields.

## 10. Conclusions

Despite the increased emphasis on integrated disease management, the use of pesticides continues to be the primary strategy for disease control in peanut cultivation, much like in other crops. However, there is a growing recognition that conventional approaches must evolve and be supplanted by modern, more effective techniques. Therefore, it is essential to consolidate research findings to understand the effectiveness, limitations, and impacts of advanced technologies used in identifying peanut diseases and applying targeted agrochemicals. This review of the literature revealed that emerging precision or variable-rate technologies offer real-time, precise, automated, and large-scale solutions for disease detection and management, assuring more effective and comprehensive coverage as compared to slow and less effective conventional techniques. The integration of smart sprayer technologies and advanced sensing methods represents a significant leap forward in agricultural disease management, as demonstrated by numerous studies conducted over the past decade. Moreover, to meet the demands of a growing population and evolving agricultural challenges, there is a pressing need for more commercial sprayers—both ground-based and UAV-based—that incorporate these advanced precision technologies. Adopting these innovative technologies is necessary to meet the growing need for sustainable and efficient agricultural practices, particularly considering the increasingly demanding market. One of the major challenges in adopting these technologies is the initial high cost. To address this adoption challenge for growers, it is necessary to develop more cost-effective, easy-to-use, and reliable technologies. This shift towards advanced methods not only enhances productivity but also aligns with environmental sustainability goals, ultimately leading to healthier crops and more resilient farming systems.

## Figures and Tables

**Table 1:** Summary of different types of ground-based disease scouting sensors from published studies.

Study Target	Purpose	Sensor	Effective Wavelength	Measuring Platform	Detection Approach and Model	AI Algorithm and Accuracy	Limitation	Ref
Peanut southern blight	Detection	ASD Field Spec3 spectrometer	350–2500 nm	Hand-held	Reflectance spectral measurement	SVM [91.8% (Kappa coeff. 0.89) on training, 62.3% (Kappa coeff. 0.43) on validation]	Incapable of discerning the intricate interplay between diseases and plants	[73]
Late leaf spot and ground nut rosette virus disease	Identify diseases	Sony α6000 digital camera, Green-Seeker crop sensor, FLIR C2 thermal camera	400 nm–700 nm	Hand-held sensor	Photogrammetry	N/A	Using three sensors separately is time-consuming, and different visual perceptions can affect the results	[67]
Peanut leaf wilting	Leaf wilting estimation	Samsung NX300 digital camera	400 nm–700 nm	Hand-held	Photogrammetry	Classification accuracy of four logistic models. Model 1: 71% Model 2: 75% Model 3: 93% Model 4: 95%		[68]
Peanut leaf spot	Early sensing	Portable field spectrometer and Fluke Ti32 thermo- vision camera	520–600 nm and 700–850 nm	Hand-held (50 cm above canopy)	Spectroscopy and thermal imaging	78% for early leaf spot and 89% for late leaf spot	Inconclusive in assessing the disease severity, especially during its initial stages	[75]
Peanut leaf spots	Detection	FieldSpec UV/VNIR Spectroradio-meter	325–1075 nm	Hand-held	Hyperspectral reflectance	N/A	Not applicable for crops in fields	[71]
Peanut leaf spots	Classification	HR2000+ high-resolution spectrometer	570 nm, 671 nm, and 750 nm	Hand-held	Hyperspectral reflectance	SVM (96.88% accuracy, Kappa Coeff. 0.95), KNN, backpropagation	Eight feature selection methods produced many feature wavelengths, which reduced the disease detection accuracy	[72]
Peanut foliar diseases	Diagnosis	Samsung Galaxy S5 and S10e, Google Pixel 3, Canon EOS Rebel T6i	400 nm–700 nm	Hand-held	Image analysis and regression modeling	Logistic regression (accuracies between 89.4 and 98.7%)	The algorithms are trained on a limited dataset that may not fully represent the diverse conditions of peanut crop growth	[69]
Peanut leaf diseases	Online detection and recognition	OLYMPUS IMAGING CORPC7070WZ camera, Sony DSC-HX30V camera and Canon EOS Kiss X5 camera	3072 × 2304 pixels 4896 × 2752 pixels 5184 × 3456 pixels	Hand-held	Machine learning	MobileNet V2: 97.8%, Xception: 99%, NasNetMobil: 97.4%. Average on-site diagnostic accuracy: >85%	The model accuracy for the classification of black spots and healthy leaves was low	[70]
Peanut stem rot	Classification	Jaz spectrometer and SpectroClip probe	200 to 1100 nm	Hand-held	Machine learning and hyperspectral reflectance	KNN, RF, SVML (Support Vector Machine Linear), PLS-DA (partial least squares discriminant analysis), GBoost, and XGBoost, NB, LDA, and MLPNN (accuracy approx. 80%)	Classification accuracy of diseased and healthy plants was low to medium	[74]

**Table 2:** Summary of different types of UAVs used for disease scouting from published studies.

Study Target	Purpose	Sensor	Effective Wavelength	Measuring Platform	Detection Approach and Model	AI Algorithms Applied and Accuracy	Limitations	Ref
Peanut leaf spot	Disease classification	GaiaSky-mini hyperspectral imager	400–1000 nm, 3.5 nm resolution	Matrice 600 UAV	Hyperspectral reflectance	KNN at field scale (90.29% and 87.04%), multinomial logistic regression (MLR) at leaf scale (93.77% accuracy and Kappa coeff. 0.91) and plant scale (92.50% accuracy and 0.89)	This study does not validate the proposed spectral indices (SIs) in multiple environments and cultivars. The SIs do not distinguish between other biotic and abiotic stresses.	[85]
Bacterial wilt	Disease-stage determination	Parrot Sequoia^®^ multispectral camera	G: 550 nm R: 660 nm RE: 735 nm NIR: 790 nm	Parrot Bluegrass UAV	Multispectral reflectance	Multi-Lasyer Perceptron (MLP) neural network for wavelength analysis	This study was only focused on leaf-level reflectance and was not conducted at the peanut canopy scale in different ecological locations and with different peanut cultivars.	[79]
Late leaf spot	Detection	MicaSense RedEdge sensor	R, G, B, NIR, and RE	Phantom 3 Advanced multi-rotor drone	Multispectral Imaging	N/A	Estimated disease only at late growth stage.	[82]
Peanut leaf spot	Disease phenotyping	Triton 5.0 MP polarized camera	N/A	DJI Inspire 2 drone	Polarized imaging	N/A	Passing clouds affect the brightness.	[87]
Tomato spot wilt disease	High-throughput phenotyping	MicaSense RedEdge multispectral camera	B: 475 nm G: 560 nm R: 668 nm NIR: 840 nm	DJI Phantom 3 quadcopter	Multispectral Imaging	N/A	Custom integration of equipment is challenging. Unreliable power regulation.	[83]

**Table 3:** Summary of different types of ground sprayers from published studies.

Study Target	Chemical Used	Sprayer Type	Performance	Limitation	Ref
Stem rot	Fungicide	CO_2_-pressurized belt pack sprayer	Varies based on multiple parameters like spray timing and type of fungicide	Results are affected by certain environmental variables, rendering them less applicable to all geographical regions	[96]
Rhizoctonia pod rot	Fungicides	Backpack sprayer	The incidence of Rhizoctonia pod rot was significantly diminished across all six cultivars following the application of tebuconazole or azoxystrobin	Particular environmental conditions and the restricted range of fungicides evaluated	[93]
Early leaf spot	Pyraclostrobin	Multiple-boom tractor-mounted CO_2_-propellant sprayer	All fungicide applications led to reduced leaf spot severity and increased yields	Fails to consider the possibility of development of resistance to fungicide	[99]
Soil-borne peanut diseases (stem rot)	Protectant fungicides	Tractor-mounted sprayer and CO_2_-pressurized belt-pack sprayer equipped with a broadcast boom	Enhanced spray coverage, density, and droplet size, particularly in the lower plant canopy, facilitate better disease management	Canopy opening are recommended for better spray penetration	[104]
Late leaf spot	Benomyl and chlorothalonil	Multiple-boom tractor-mounted CO_2_-propellant sprayer	Combining benomyl with chlorothalonil yields better disease control compared to benomyl use alone	Resistance to benomyl has emerged in pathogen populations	[100]
Late leaf spot	Plant growth-promoting rhizobacteria (PGPR) and chemical elicitors	CO_2_ backpack sprayer	PGPR was shown to be effective in eliciting systemic resistance in multiple crops	The study fails to find if chemical elicitors can induce systemically acquired resistance in peanuts	[92]
Sclerotinia blight	Fungicide	Self-propelled boom sprayer	The flat-fan nozzles delivered a high spray volume (>360 l/ha) compared to the hollow-cone nozzles, which delivered 206 l/ha	Intense precipitation may postpone the application of fungicides by tractor-mounted sprayers	[98]

**Table 4:** Summary of different types of UAV-based sprayers from published studies, including spray application parameters, performance metrics, control effects, applicable scenarios, and performance.

UAV and Crop	Spray Application Parameters (1) Tank Capacity (Litre) (2) Spray Vol. (Litre/ha)	Operational Efficiency (1) Flight Speed (m/s) (2) Swath Width (m) (3) Flight Height (m)	Control Effects	Applicable Scenarios	Performance	Ref.
T20 plant protection, Peanut	(1) 20 (2) 22.5	(1) 5 m/s (2) 5 m (3) 2 m	Disease control (leaf spot and rust): Droplet density (droplets/cm^2^) Canopy: 25.7–28.2 Ground: 3.2–3.3	Peanut fields Applications requiring optimized pesticide usage	UAV spray treatment at 22.5 L ha^−1^ showed higher control over peanut defoliation, yield, leaf spot, and rust, with just 0.3% less effectiveness in leaf spot control, compared to an electric knapsack sprayer.	[111]
EA-20X, Peanut	(1) N/A (2) 15	(1) 3 (2) N/A (3) 2	Pesticide deposition on peanut plants: OD-50 * had double the pesticide retention compared to SC-50 *. MO501 at 0.1% increased deposition by ≈40%. Silwet 408 and XL-70 (0.1%) reduced deposition by >50%.	Peanut plants Applicable for UAV-based low-dilution pesticide application Particularly beneficial for hydrophobic leaf surfaces	OD-50 formulation showed the highest pesticide retention. MO501 (0.1%) improved efficiency but did not completely prevent drift. Silwet 408 and XL-70 promoted spreading but increased spray drift. OD-50 had the lowest fine droplet fraction, reducing drift and increasing pesticide efficiency.	[107]
N-3-type helicopter, Maize	(1) 25 (2) 0.6–1	(1) 3 (2) 7 (3) 5 and 7	At 5 m flight height, the dispersion value was 0.17. At 7 m flight height, the dispersion value was 0.10. A higher droplet deposition was observed in the upper and ear layers of the maize canopy compared to the top and bottom layers.	Suitable for late-stage maize growth where canopy density is high	Optimal droplet deposition and uniformity at a spraying swath of 7 m with a deposition percentage of 38.4% for a 5 m swath and 38.1% for a 9 m swath.	[120]
Single-rotor HyB-15L, Rice	(1) 15 (2) 15	(1) 3–5 (2) 4–5 (3) 0.8–1.5	Efficacy against brown plant hopper (BPH) after spraying (% mortality): 3 days: 90.3 5 days: 87 10 days: 78.6	Rice fields with dense canopy layers Pest control in the late growth stage of rice, where plant hoppers are prevalent	Insecticidal efficacy of 92–74% over 10 days	[121]
P-20 Quadrotor, Rice	(1) 6 (2) N/A	(1) 3.7 (2) N/A (3) 2	The highest deposition achieved was 68.69%. The most significant influence factor was flight height, followed by flight speed and then nozzle flow rate.	Applicable to varied UAV agricultural spraying operations	The optimal combination of parameters, including nozzle flow rate at 430 mL/min, achieved a droplet deposition level on target of 68.69%. This indicated a high level of effectiveness in applying pesticides, with the relative error in the model being less than ±5%.	[122]
Jifei P20 Quadrotor, Cotton	(1) 6, 8, 10 (2) N/A	(1) 1–8 (2) 1.5–3 (3) 1.5 and 2	Flight height significantly affected droplet deposition and drift. Better droplet deposition and uniformity at 2.0 m compared to 1.5 m.	Precision spraying in cotton farms, especially in large-scale production areas	Control efficiencies of 63.7% for cotton aphids and 61.3% for spider mites, with slightly lower pest control and leaf absorption compared to boom spraying.	[115]
Six-rotor V6A, N/A	(1) 5 (2) N/A	(1) 7 (2) 5–7 (3) 3	Higher pressure (517 kPa) produced finer droplets, which increased drift potential.	Ideal for targeted applications like edge spraying and spot spraying Application of granular products like mosquito control	N/A	[123]
Eight-rotor MG-1, N/A	(1) 10 (2) N/A	(1) 3 (2) 5–7 (3) 2	A higher flow rate (354 mL/min) led to larger droplets, improving canopy penetration.	Efficient for crops needing better droplet penetration It can be optimized for drift-sensitive environments	N/A	[123]
N-3 helicopter, Wheat	(1) 25 (2) N/A	(1) 4 (2) 7 (3) 5	At 5.0 m, droplet coverage on the lower layer was 45.6% of the upper layer. Pest control efficiency after 10 days of application using 450 g/ha: 72.89% disease control.	Effective in wheat fields with dense canopy layers Useful for disease control during the wheat heading stage	UAV sprayer achieved a droplet coverage rate of 45.6% on the wheat lower layer and a control efficiency of 55.1% against powdery mildew, with performance comparable to a knapsack-type electric sprayer at a reduced dosage.	[114]

* OD-50: Oil dispersion treatment, SC-50: suspension concentrate.

**Table 5:** Summary of different types of commercial precision sprayers in pesticide management of different crops from published studies of the past decade.

Sprayer (Name)	Platform (Ground-Based/ UAV-Based)	Sensors Integrated	Tank Capacity (gal)	Spray Speed (mph)	Price (USD)	Performance	Ref.
See & Spray™ Select	Ground-based	RGB camera for green plant detection, John Deere ExactApply™ nozzle control	1600, 1200, 1000	12	~25,000	77% reduction in herbicide use on average compared to broadcast spraying	[127]
John Deere R4038	Ground-based	John Deere Section control	1000	0–25	~295,000	High efficiency and precise application	[128]
Case IH Patriot 4440	Ground-based	AFS AccuGuide, AIM Command FLEX, AutoBoom	1200	0–30	~350,000	Uniform spray distribution, Precise and flexible control	[129]
Hardi Saritor 5000	Ground-based	SprayCenter control, HC 9600 display	1320	2–18	~300,000	Broad spraying, automatic operating speed	[130]
Horsch Leeb PT 330	Ground-based	BoomControl	1585	18	~380,000	Excellent maneuverability, Automotive drive control	[131]
Yamaha RMAX	UAV-based	Thermo unit sensor, RADAR	4	13	~100,000	Precise and targeted spraying, high maneuverability	[132]
DJI Agras T20	UAV-based	Real-time kinematic GPS	5	14.5	~14,999	Autonomous high-precision operation	[126]
DJI Agras T40	UAV-based	Active Phased Array Radar+, binocular vision	10.5	22	~16,000–26,000	Higher payload capacity; supports multiple missions like spraying, mapping and surveying	[133]

## Abbreviations

The following abbreviations are used in this manuscript:

AI	Artificial intelligence
ANN	Artificial neural network
ASD	Analytical spectral device
BP	Backpropagation
BWI	Bacterial Wilt Index
CNN	Convolutional neural network
ELISA	Enzyme-linked immunosorbent assay
eVTOL	Electric vertical takeoff and landing
ExG	Excess green
FAA	Federal Aviation Administration
FLIR	Forward-looking infrared
GNDVI	Green Normalized Difference Vegetation Index
GPS	Global Positioning System
HCI	Hope Color Index
HTP	High-throughput phenotyping
KNN	K-Nearest Neighbors
LAI	Leaf Area Index
LDA	Linear discriminant analysis
MLS	Mobile laser scanning
MLR	Multinomial logistic regression
NIR	Near-infrared
NDRE	Normalized Difference Red Edge
NDVI	Normalized Difference Vegetation Index
NPK	Nitrogen, phosphorus, potassium
PGPR	Plant growth-promoting rhizobacteria
PCR	Polymerase chain reaction
PLSDA	Partial least squares discriminant analysis
RGB	Red–green–blue
REVI	Reduced Simple Ratio Vegetation Index
RF	Random Forest
SI	Spectral index
SOR	Statistical outlier removal
SR	Simple Ratio
SVM	Support Vector Machine
TLS	Terrestrial laser scanning
UAV	Unmanned aerial vehicle
USDA	United States Department of Agriculture
VNIR	Visible near-infrared
WSN	Wireless sensor network
XGBoost	Extreme Gradient Boosting
